# Transforming growth factor beta type 1 (TGF-β) and hypoxia-inducible factor 1 (HIF-1) transcription complex as master regulators of the immunosuppressive protein galectin-9 expression in human cancer and embryonic cells

**DOI:** 10.18632/aging.202343

**Published:** 2020-12-08

**Authors:** Anette Teo Hansen Selnø, Stephanie Schlichtner, Inna M. Yasinska, Svetlana S. Sakhnevych, Walter Fiedler, Jasmin Wellbrock, Elena Klenova, Ludmila Pavlova, Bernhard F. Gibbs, Martin Degen, Isabelle Schnyder, Nijas Aliu, Steffen M. Berger, Elizaveta Fasler-Kan, Vadim V. Sumbayev

**Affiliations:** 1 Medway School of Pharmacy, Universities of Kent and Greenwich, Chatham Maritime, United Kingdom; 2 Department of Oncology, Hematology and Bone Marrow Transplantation with Section Pneumology, Hubertus Wald University Cancer Center, University Medical Center Hamburg-Eppendorf, Hamburg, Germany; 3 School of Biological Sciences, University of Essex, Colchester, United Kingdom; 4 Division of Experimental Allergology and Immunodermatology, University of Oldenburg, Oldenburg, Germany; 5 Dental Research Center, Department of Orthodontics and Dentofacial Orthopedics, University of Bern, Bern, Switzerland; 6 Department of Pediatric Surgery, Children’s Hospital, Inselspital Bern, University of Bern, Bern, Switzerland; 7 Department of Biomedicine, University of Basel and University Hospital Basel, Basel, Switzerland; 8 Department of Biomedical Research, University of Bern, Bern, Switzerland; 9 Department of Human Genetics, Children’s Hospital, Inselspital, University of Bern, Bern, Switzerland

**Keywords:** galectin-9, immune escape, TGF-beta, HIF-1

## Abstract

Galectin-9 is one of the key proteins employed by a variety of human malignancies to suppress anti-cancer activities of cytotoxic lymphoid cells and thus escape immune surveillance. Human cancer cells in most cases express higher levels of galectin-9 compared to non-transformed cells. However, the biochemical mechanisms underlying this phenomenon remain unclear.

Here we report for the first time that in human cancer as well as embryonic cells, the transcription factors hypoxia-inducible factor 1 (HIF-1) and activator protein 1 (AP-1) are involved in upregulation of transforming growth factor beta 1 (TGF-β1) expression, leading to activation of the transcription factor Smad3 through autocrine action. This process triggers upregulation of galectin-9 expression in both malignant (mainly in breast and colorectal cancer as well as acute myeloid leukaemia (AML)) and embryonic cells. The effect, however, was not observed in mature non-transformed human cells. TGF-β1-activated Smad3 therefore displays differential behaviour in human cancer and embryonic *vs* non-malignant cells. This study uncovered a self-supporting biochemical mechanism underlying high levels of galectin-9 expression operated by the human cancer and embryonic cells employed in our investigations. Our results suggest the possibility of using the TGF-β1 signalling pathway as a potential highly efficient target for cancer immunotherapy.

## INTRODUCTION

Galectin-9 is one of the crucial proteins used by various types of cancer cells to suppress cytotoxic immune responses and thus, escape immune surveillance [1]. Some cancer cells (acute myeloid leukaemia (AML) and colorectal cancer) are capable of secreting this protein, while other cancer cells translocate galectin-9 onto the surface [1] and use it to impair anti-cancer activities of cytotoxic lymphoid cells such as cytotoxic T lymphocytes and natural killer (NK) cells [1–6]. Galectin-9 lacks a secretion signal sequence and thus cannot be secreted on its own. Its receptor, the T cell immunoglobulin and mucin domain containing protein 3 (Tim-3), can also act as a possible trafficker for galectin-9 [7]. When complexed with Tim-3 on the cell surface, galectin-9 induces downstream signalling of differential intensity [8–10], depending on the type of human myeloid and lymphoid cells [11]. In myeloid cells, galectin-9 primarily triggers growth factor type responses, while in lymphoid cells it induces pro-apoptotic signalling [10–13]. Galectin-9, together with Tim-3, can be shed from the cell surface by proteolytic enzymes, thus being released into the tumour microenvironment or blood [2].

Human cancer cells express significantly higher levels of galectin-9 compared to healthy human cells [1]. In particular, high amounts of galectin-9 are secreted by AML and colorectal cancer cells [1, 14]. However, the biochemical mechanisms underlying increased galectin-9 expression in human cancer cells are unknown. Understanding these mechanisms will significantly improve our knowledge concerning the biochemistry of malignant tumour immune escape and would facilitate identification of new targets for efficient cancer immunotherapy.

It has been reported that human cancer cells produce transforming growth factor beta type 1 (TGF-β1, also known as TGF-β), that can display autocrine activity by binding to TGF-β receptors (TGFBR) [15, 16]. TGF-β is known to transduce its signal *via* the Smad3 transcription factor, which triggers the expression of target genes [17]. The galectin-9 gene LGALS9 promoter region has several (at least 5) Smad3 response elements and Smad3 has been reported to induce galectin-9 expression [18, 19]. In addition, the TGF-β encoding gene has at least 9 Smad3 response elements in its promoter region and thus could also upregulate TGF-β expression in an autocrine manner capable of supporting itself without external signals. Initial activation of TGF-β could be induced by the hypoxia-inducible factor 1 (HIF-1) transcription complex, which contains two subunits – a constitutive β subunit and an inducible α subunit. HIF-1 displays high activity in the early stages of tumour growth and thus could initiate TGF-β expression [20], which can then trigger the autocrine pathway described above leading to galectin-9 overexpression. In addition, the activities of enzymes which generate reactive oxygen species (ROS), such as NADPH oxidase and xanthine oxidase, are elevated in cancer cells compared to healthy cells in corresponding tissues [20, 21]. Increased ROS levels lead to the activation of apoptosis signal regulating kinase 1 (ASK1) and its downstream pathway, resulting in activation of the AP-1 (activator protein 1) [22] transcription complex which could upregulate TGF-β expression.

Importantly, similar events could also occur in human embryonic cells, thus leading to the expression of high levels of galectin-9 and preventing embryo rejection by mother’s immune system [23]. Experimental investigation of this complex hypothesis became the aim of this study.

Here we report for the first time that in human breast cancer, AML and embryonic cells, HIF-1 and AP-1 upregulate the expression of TGF-β, leading to the activation of Smad3 through autocrine action. This process subsequently upregulates galectin-9 expression in both malignant and embryonic cells, but not in mature healthy human cells. Activated Smad3 therefore displays differential behaviour in cancer/embryonic *vs* healthy cells.

## RESULTS

### HIF-1, TGF-β, Smad3 and galectin-9 are highly upregulated in primary human cancer and embryonic cells

In order to investigate the hypothesis of self-sustaining upregulation of TGF-β and galectin-9 expression in human cancer and embryonic cells we tested primary human breast tumours, primary AML cells as well as primary embryonic cells.

**Figure 1:**
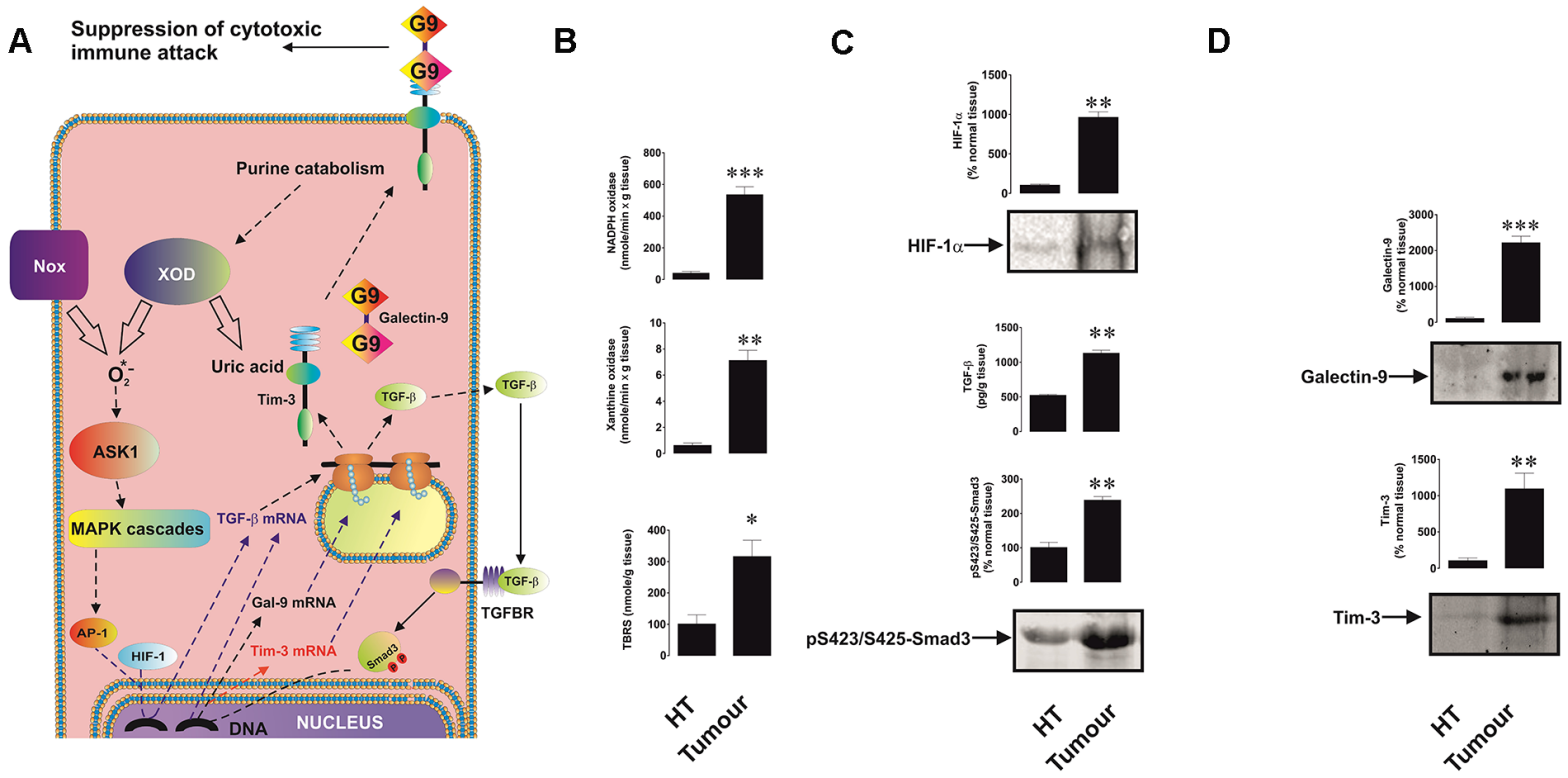
**Increased redox status, upregulated HIF-1α and TGF-β/Smad3 pathways as well as Tim-3 and galectin-9 expression in breast tumour tissues compared to non-transformed peripheral tissues.** The proposed pathway studied is summarised in panel (**A**), where it is indicated that xanthine oxidase (XOD) and NADPH oxidase (Nox) produce ROS which activate AP-1 transcription factor through ASK1-controlled MAP kinase cascades. HIF-1 and AP-1 contribute to the activation of TGF-β expression, which then displays autocrine activity and stimulates the activation of galectin-9 and possibly Tim-3 expression through Smad3 transcription factor. Tissue lysates were subjected to measurement of xanthine oxidase and NADPH oxidase activities as well as TBRS levels (**B**). HIF-1α accumulation, tissue-associated TGF-β and phospho-S423/S425-Smad3 levels (**C**) as well as levels of tissue-associated Tim-3 and galectin-9 (**D**) were analysed in tissue lysates. All quantities are expressed in respective units per 1 gram of the tissue. Normalisations against total protein loaded (for Western blot; measured by Li-Cor protein assay kit) and per mg of the total protein for enzyme activities and TBRS assays were also performed. These results are presented in the Supplementary Figure 1. Images are from one experiment representative of five which gave similar results. Data are shown as mean values ± SEM of five independent experiments. * - p < 0.05 and ** - p < 0.01 *vs* non-transformed peripheral tissue abbreviated as HT (healthy tissue).

We found that all five tested breast cancer patients showed very high levels of xanthine oxidase and NADPH oxidase activities as well as thiobarbiturate-reactive species (TBRS, products of lipid peroxidation indicating increased oxidative burst) levels in tumour tissues compared to healthy tissues (Figure 1A, 1B). Respectively, levels of HIF-1α were also significantly higher in tumour samples (Figure 1C). This was in line with the highly increased amounts of tumour-associated TGF-β and intracellular levels of phospho-S423/S425-Smad3 (active form, Figure 1C). In line with our previous observations [1], levels of both Tim-3 and galectin-9 were substantially increased in tumour tissues compared to non-malignant samples (Figure 1D).

**Figure 2:**
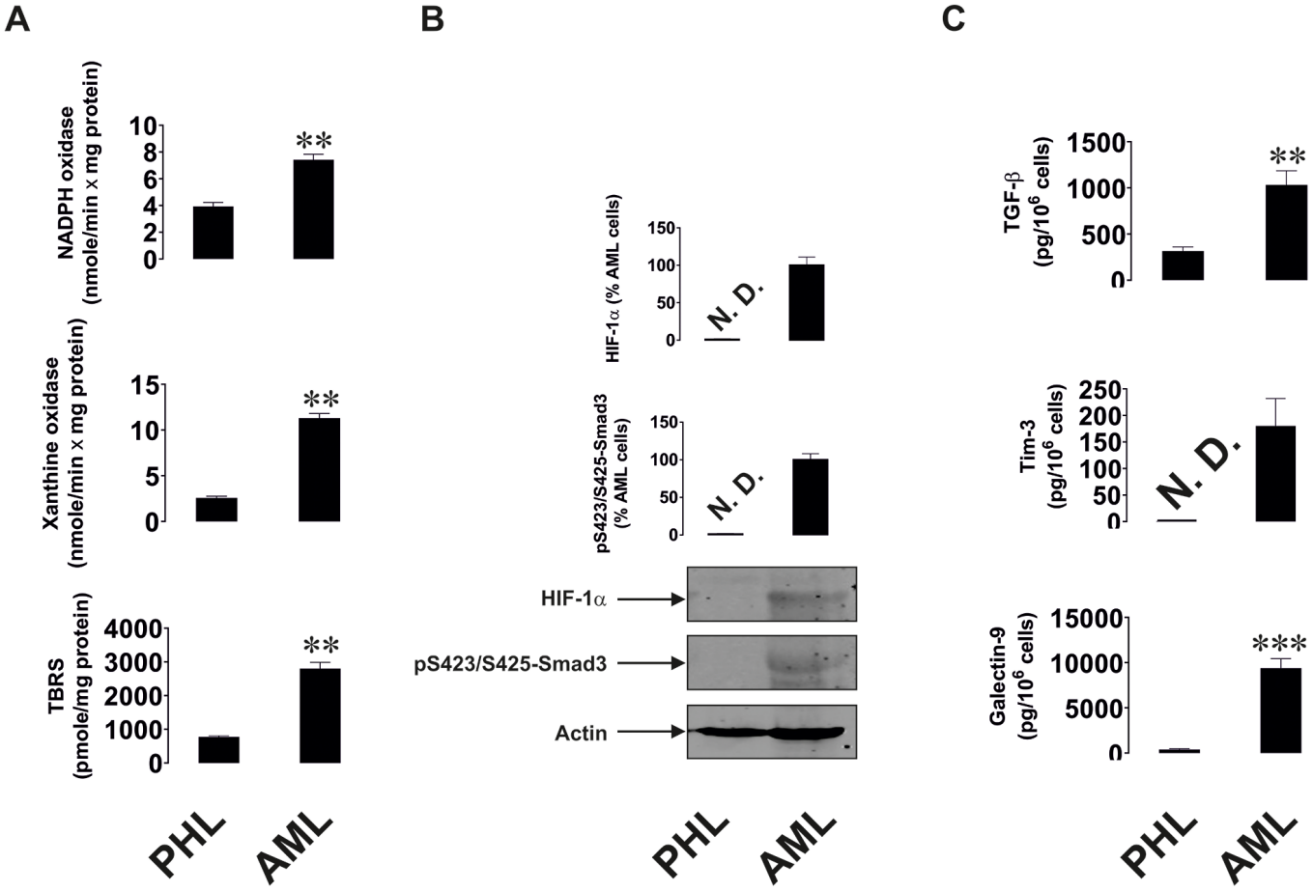
**Increased redox status, upregulated HIF-1α and TGF-β/Smad3 pathways as well as Tim-3 and galectin-9 expression in primary human AML cells compared to non-transformed mononuclear leukocytes.** Measurements were conducted in primary human AML cells *vs* primary mononuclear leukocytes obtained from healthy donors. Activities of xanthine oxidase, NADPH oxidase and TBRS levels (**A**). Levels of accumulated HIF-1α protein and phospho-S423/S425-Smad3 (**B**). Levels of secreted TGF-β, Tim-3 and galectin-9 measured in cell culture medium (**C**). Images are from one experiment representative of five which gave similar results. Data are shown as mean values ± SEM of five independent experiments. * - p < 0.05 and ** - p < 0.01 *vs* non-transformed (“healthy”) primary human mononuclear leukocytes (abbreviated as PHL – “primary healthy leukocytes”).

Similar observations were seen in AML, a non-solid malignancy. AML cells isolated from newly diagnosed patients were compared with primary leukocytes isolated from healthy donors upon culturing them for 24 h. AML cells showed significantly upregulated xanthine oxidase and NADPH oxidase activities as well as TBRS levels (Figure 2A), which suggests a higher level of oxidative stress in AML cells. HIF-1α and phospho-S423/S425-Smad3 were almost undetectable in primary healthy leukocytes, but were clearly detectable in AML cells (Figure 2B). AML cells released significantly higher amounts of TGF-β compared to healthy leukocytes. Respectively, AML cells secreted much higher amounts of both Tim-3 and galectin-9 (Figure 2C; secreted levels were measured since over 24 h AML cells release much higher amounts of these proteins compared to those present in the cells at a single moment of time, when the cells are harvested).

**Figure 3:**
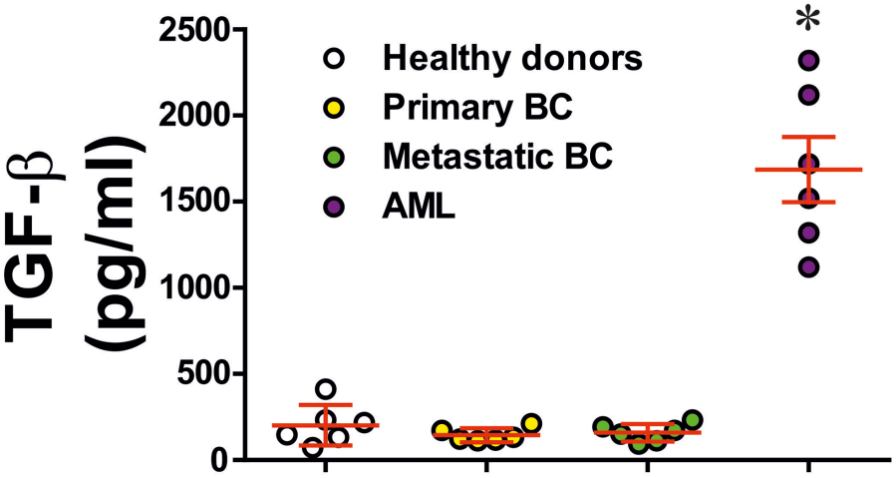
**Levels of secreted TGF-β in blood plasma of healthy donors, primary and metastatic breast cancer patients and AML patients.** TGF-β concentrations were measured in blood plasma of healthy donors, patients with primary breast tumours, patients with metastatic breast solid tumours and AML patients (n=6 for all donor types). Data are shown as mean values ± SEM (data for each patient are shown). * - p < 0.05 *vs* healthy donors.

To understand the role of TGF-β we analysed blood plasma of six healthy donors, six primary breast cancer patients, six metastatic breast cancer patients and six AML patients. In cases of primary and metastatic breast cancers, blood plasma levels of TGF-β were similar to those in healthy donors. However, in AML patients they were strikingly and significantly elevated (Figure 3). These results suggest that in solid tumours, like primary and metastatic breast tumours, produced TGF-β most likely remains in the tumour microenvironment while in the case of AML, this growth factor is secreted into the peripheral blood and can be employed by circulating AML cells.

**Figure 4:**
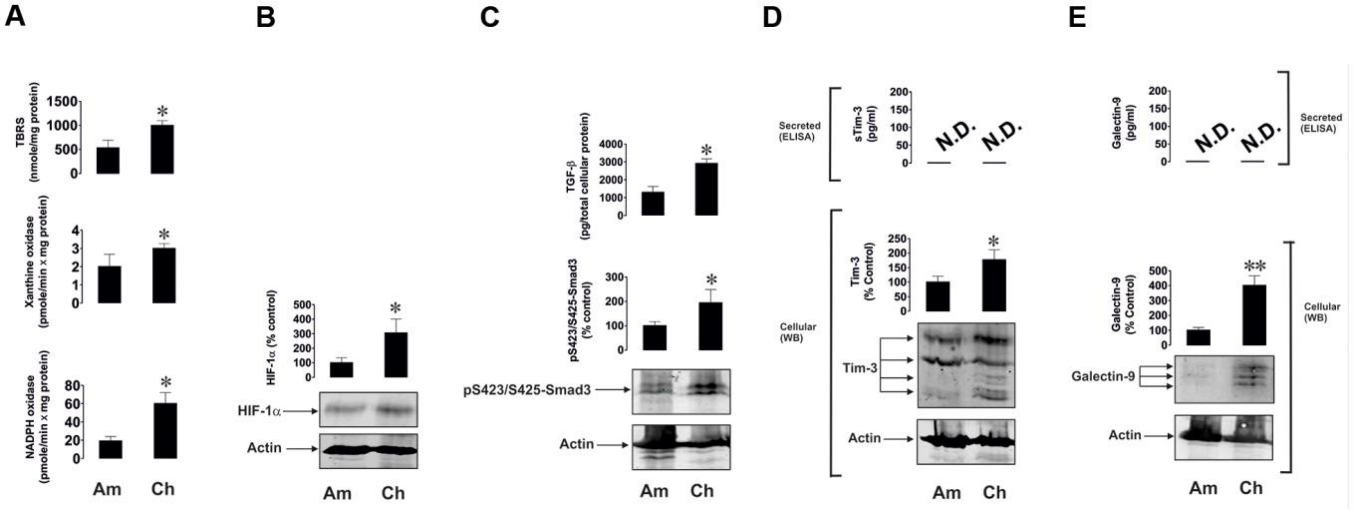
**Oxidative burst, HIF-1α accumulation, TGF-β/Smad3 pathway and Tim-3/galectin-9 levels are highly upregulated in primary human embryonic cells at early pregnancy stages.** Primary human embryonic cells, obtained from amniotic fluid (Am, around 20 - 25 weeks of pregnancy) and chorion (Ch, around 13 weeks of pregnancy), were subjected to measurement of xanthine oxidase and NADPH oxidase activities as well as TBRS levels (**A**). HIF-1α accumulation (**B**), secreted TGF-β and cell-associated phospho-S423/S425-Smad3 levels were also analysed (**C**), as well as levels of cell-associated and secreted Tim-3 (**D**) and galectin-9 (**E**). Images are from one experiment representative of seven, which gave similar results. Data are shown as mean values ± SEM of seven independent experiments. * - p < 0.05 *vs* amniotic cells.

Intriguingly, primary human embryonic cells obtained from chorion (around week 13 of pregnancy) of seven pregnant patients and amniotic liquid obtained from another seven patients (between weeks 20 and 25) had clearly detectable activities of xanthine oxidase, NADPH oxidase and TBRS (Figure 4A). The earlier the stage was, the higher was the level of oxidative burst. A similar pattern was observed for HIF-1α, secreted TGF-β and phospho-S423/S425-Smad3 levels (Figure 4B, 4C). Respectively, Tim-3 and galectin-9 were clearly detectable in both cell types, although not secreted, and were significantly higher at earlier pregnancy stages (Figure 4D, 4E).

### Redox-dependent mechanisms contribute to TGF-β and galectin-9 expression

**Figure 5:**
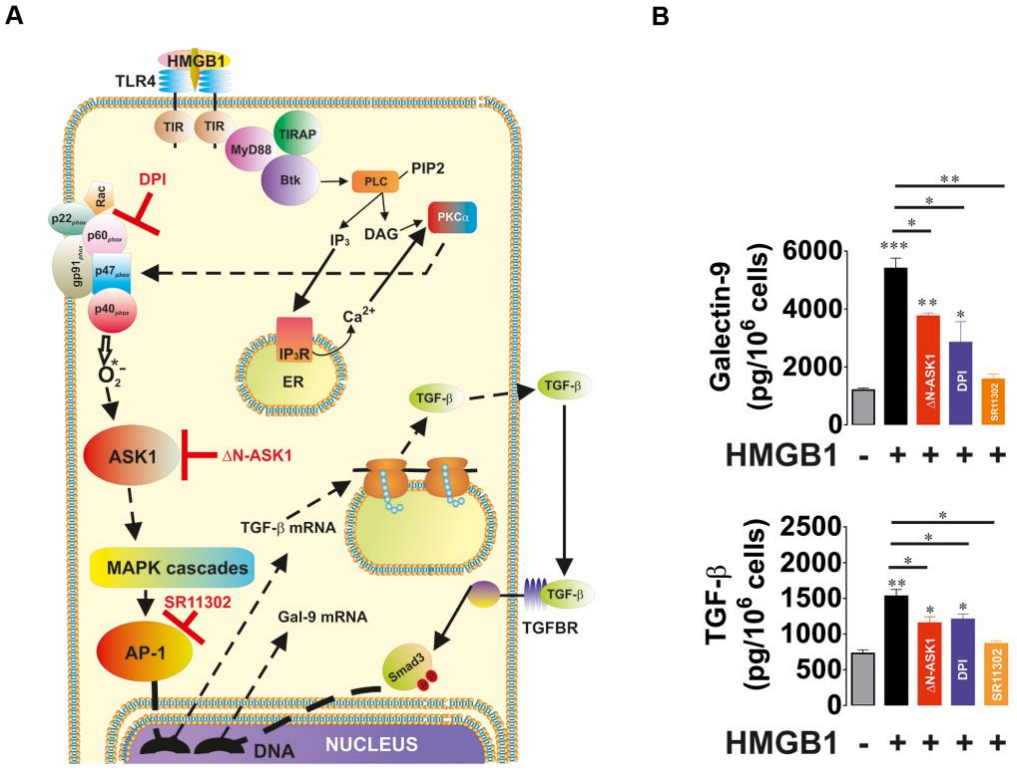
**Oxidative stress-induced activation of AP-1 in an ASK1-dependent manner induces TGF-β and galectin-9 expression.** THP-1 cells were treated with the Toll-like receptor 4 (TLR4) ligand, high mobility group box 1 (HMGB1), for 24 h. TLR4 mediates activation of NADPH oxidase using myeloid differentiation factor 88 (MyD88), TLR4 TIR domain-associated protein (TIRAP) and Bruton’s tyrosine kinase (Btk). Activation of Btk by MyD88 and TIRAP leads to Btk-dependent phosphorylation of phospholipase C (PLC, mainly isoform 1γ), which triggers activation of protein kinase C alpha (PKCα). PKCα activates NADPH oxidase which produces superoxide anion radical, activating the ASK1 pathway and activation of AP-1 transcription factor. The scheme is shown in panel (**A**). THP-1 cells were exposed for 24 h to 1 μg/ml HMGB1 with or without pre-treatment with 30 μM DPI (NADPH oxidase inhibitor), 1 μM SR11302 (AP-1 inhibitor) or transfection with dominant-negative isoform of ASK1 (ΔN-ASK1). Levels of secreted TGF-β and galectin-9 were measured by ELISA (**B**). Data are shown as mean values ± SEM of four independent experiments. * - p < 0.05 and ** - p < 0.01 *vs* control.

In order to understand the ability of redox-dependent ASK1-mediated activation of AP-1 in TGF-β and galectin-9 production, we used THP-1 human acute myeloid leukaemia cells which express Toll-like receptor 4 (TLR4; Figure 5A). Cells were exposed for 24 h to 1 μg/ml high mobility group box 1 (HMGB1) protein. We found that HMGB1 induced the secretion of TGF-β and galectin-9 by THP-1 cells (Figure 5B). To investigate the contribution of the NADPH oxidase–ASK1–AP-1 redox-dependent pathway to TGF-β expression we pre-treated the cells with 30 μM DPI (Diphenyleneiodonium chloride, NADPH oxidase inhibitor) or 1 μM SR11302 (AP-1 inhibitor) for 1 h before exposing them for 24 h to HMGB1. Another set of cells was subjected to transfection with dominant-negative ASK1 (ΔN-ASK1), to block the activity of this enzyme prior to the 24 h exposure to HMGB1. We found that HMGB1 induced TGF-β and galectin-9 secretion (Figure 5B). DPI, SR11302 and ΔN-ASK1 attenuated the effect, suggesting that redox-induced ASK-1-mediated AP-1 activation leads to increased TGF-β and galectin-9 production by THP-1 cells.

**Figure 6:**
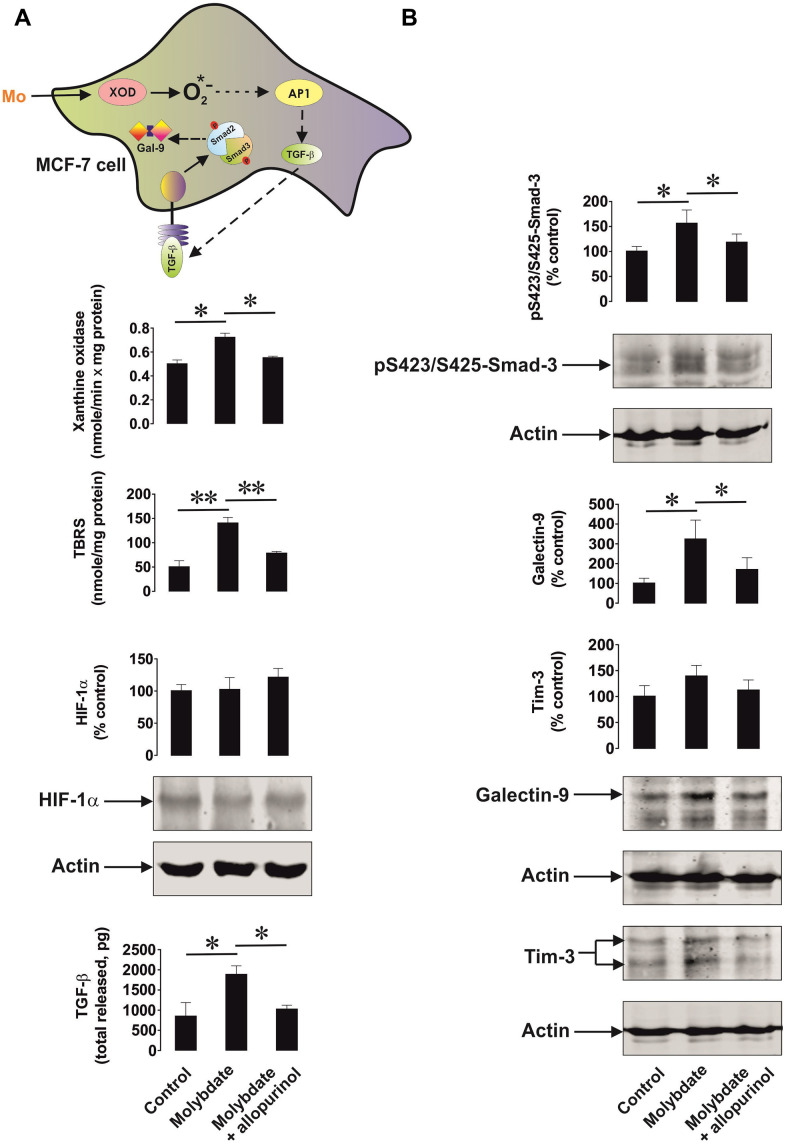
**Xanthine oxidase activation leads to increased oxidative stress and upregulation of the TGF-β/Smad3 pathway as well as galectin-9 expression.** MCF-7 human breast cancer cells were exposed to ammonium molybdate for 24 h to induce xanthine oxidase activity in the absence or presence of the xanthine oxidase inhibitor allopurinol. Xanthine oxidase activity, TBRS levels, HIF-1α accumulation, secreted TGF-β (**A**), and cell-associated phospho-S423/S425-Smad3, Tim-3 and galectin-9 (**B**) were analysed as outlined in the Materials and Methods. Images are from one experiment representative of four which gave similar results. Data are shown as mean values ± SEM of four independent experiments. * - p < 0.05 and ** - p < 0.01 *vs* indicated events.

We then studied the ability of xanthine oxidase to upregulate TGF-β production. We used MCF-7 breast cancer cells, which express xanthine oxidase [21] and induced its activity by ammonium molybdate. Xanthine oxidase is a molybdenum-containing enzyme, so excess of molybdenum converts all the available xanthine oxidase molecules into their active form. To confirm the specificity of the effect we exposed MCF-7 cells to 100 μg/ml ammonium molybdate for 24 h in the absence or presence of 250 μg/ml allopurinol, a specific xanthine oxidase inhibitor. We found that xanthine oxidase activity was significantly upregulated by molybdate in MCF-7 cells (Figure 6A). This led to increased oxidative burst based on significantly increased TBRS levels. Xanthine oxidase activation was not able to induce HIF-1α accumulation but the level of secreted TGF-β was significantly increased (Figure 6A). This resulted in a significant upregulation of Smad3 S423/S425 phosphorylation (Figure 6B). As a result, galectin-9 expression was also significantly increased (Figure 6B). Allopurinol attenuated all these effects (Figure 6A, B), indicating a specific role for xanthine oxidase in the processes outlined above.

### The HIF-1 transcription complex triggers galectin-9 expression by inducing TGF-β production; Smad3 is involved in both TGF-β and galectin-9 expression

**Figure 7:**
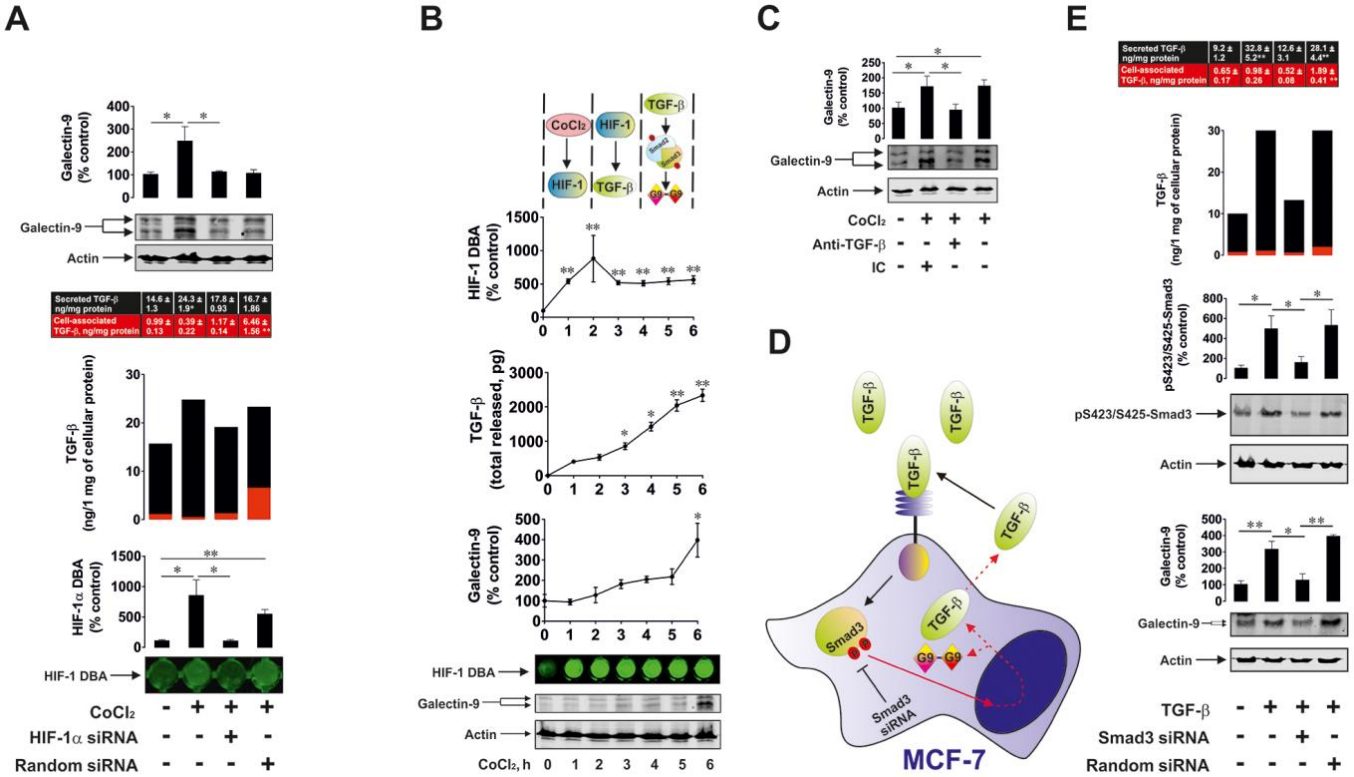
**HIF-1 and Smad3 are involved in the production of TGF-β and galectin-9.** (**A**) *Cobalt chloride induces HIF-1 activation, TGF-β and galectin-9 production.* Wild type, HIF-1α knockdown and random siRNA-transfected MCF-7 cells were exposed to 50 μM cobalt chloride for 6 h followed by measurement of HIF-1 DBA, secreted (in cell culture medium) and cell-associated (in cell lysates) TGF-β as well as cell-associated galectin-9. Images are from one experiment representative of three which gave similar results. (**B**) *Dynamics of cobalt chloride-induced HIF-1 activation, TGF-β and galectin-9 accumulation in MCF-7 human breast cancer cells.* MCF-7 cells were exposed to 50 μM cobalt chloride for 1, 2, 3, 4, 5 and 6 h followed by measurement of HIF-1 DBA, secreted and cell-associated TGF-β as well as cell-associated galectin-9. Images are from one experiment representative of three which gave similar results (in the case of TGF-β – *vs* 1 h time-point since at zero point cells can’t release any TGF-β. At this time-point, fresh medium has just been supplied and measurement was taken immediately to confirm zero TGF-β level). (**C**) *HIF-1-induced galectin-9 expression is mediated by TGF-β*. MCF-7 cells were exposed to 50 μM cobalt chloride for 6 h with or without presence of TGF-β neutralising or isotype control antibody. Galectin-9 expression was then assessed by Western blot. Images are from one experiment representative of three, which gave similar results. (**D**), (**E**) *Smad3 is involved in TGF-β and galectin-9 expression*. (**D**) Scheme of the experiment performed showing studied effects. (**E**) Wild type, Smad3 knockdown and random siRNA-transfected MCF-7 cells were exposed to 2 ng/ml TGF-β for 24 h followed by measurement of secreted (in cell culture medium) and cell-associated (in cell lysates) TGF-β as well as cell-associated galectin-9 and phospho-S423/S425 Smad3. Images are from one experiment representative of three, which gave similar results. All quantitative data are shown as mean values ± SEM (n=3-4) * - p < 0.05 and ** - p < 0.01 *vs* indicated events.

We then considered the effect of HIF-1 activation on TGF-β production and its subsequent effect on galectin-9 expression. We exposed wild type and HIF-1α knockdown (achieved by transfection of HIF-1α siRNA) MCF-7 cells as well as those transfected with random siRNA to 50 μM cobalt chloride (CoCl_2_) for 6 h (Co cations directly inhibit degradative hydroxylation of HIF-1α [24]), followed by measurement of HIF-1 DNA-binding activity, cell-associated and secreted TGFβ (ELISA) as well as cellular galectin-9 levels (Western blot – MCF-7 cells do not secrete galectin-9). We found that CoCl_2_ induced significant upregulation of HIF-1 DNA-binding activity in wild type and random siRNA-transfected MCF-7 cells (Figure 7A). No effect was observed in HIF-1α knockdown cells (Figure 7A). In wild type cells CoCl_2_ induced significant upregulation of secreted and total levels of TGF-β. The effect was not observed in HIF-1α knockdown cells (Figure 7A). In MCF-7 cells transfected with random siRNA, the level of total TGF-β was upregulated to the one observed in wild type cells exposed to CoCl_2_. However, the application of DOTAP to transfect these cells with random siRNA together with CoCl_2_ slowed down the process of TGF-β secretion. As a result, the level of galectin-9 was only upregulated in wild type MCF-7 cells treated with CoCl_2_ (Figure 7). These results suggest that HIF-1 induces the expression of TGF-β, which then facilitates the upregulation of galectin-9 expression. To further investigate this assumption we studied the dynamics of the process. We exposed wild type MCF-7 cells to 50 μM CoCl_2_ for 1, 2, 3, 4, 5 and 6 h time points and detected HIF-1 DNA-binding activity, levels of secreted TGF-β and cellular galectin-9 expressions. We found that HIF-1 DNA-binding activity was increased after 1 h of exposure to CoCl_2_, while the levels of secreted TGF-β were significantly increased following 3 h of exposure (Figure 7B). Cellular galectin-9 level was significantly upregulated only in 6 h of exposure to CoCl_2_ (Figure 7B). To specifically confirm the contribution of TGF-β in regulating galectin-9 expressions, we exposed wild type MCF-7 cells to 50 μM CoCl_2_ for 6 h in the absence or presence of TGF-β neutralising antibody or isotype control antibody. We found that TGF-β neutralising antibody but not the isotype control attenuated CoCl_2_-induced galectin-9 upregulation in MCF-7 cells (Figure 7C). All these findings clearly demonstrated that HIF-1 induces TGF-β production which displays autocrine activity and triggers galectin-9 expression.

In order to confirm the role of Smad3 in both TGF-β and galectin-9 expression we used wild type and Smad3 knockdown MCF-7 cells. As a control for reagents, we used MCF-7 cells transfected with random siRNA as outlined in Materials and Methods. Cells were exposed to 2 ng/ml TGF-β for 24 h and cell-associated (in cell lysates) and the levels of secreted (in cell culture medium) TGF-β were measured by ELISA. Phospho-S423/S425-Smad3 and galectin-9 were measured in cell lysates to confirm successful knockdown and to assess the effects on galectin-9 expression (Figure 7D). We found that the presence of TGF-β led to an increase in secreted TGF-β levels (Figure 7E). This increase did not take place in Smad3 knockdown cells. The same was applicable to the levels of galectin-9 (Figure 7E). MCF-7 cells transfected with random siRNA displayed increased levels of cell-associated as well as secreted TGF-β. This resulted in upregulation of galectin-9 expression as well (Figure 7E). However, MCF-7 cells transfected with random siRNA in the presence of CoCl_2_ displayed higher levels of cell-associated TGF-β and lower levels of secreted protein compared to similar cells treated with TGF-β. This means that the presence of DOTAP reagent and cobalt cations reduces the ability of MCF-7 cells to secrete *de novo* produced TGF-β.

### TGF-β induces galectin-9 expression in human cancer and embryonic cells

**Figure 8:**
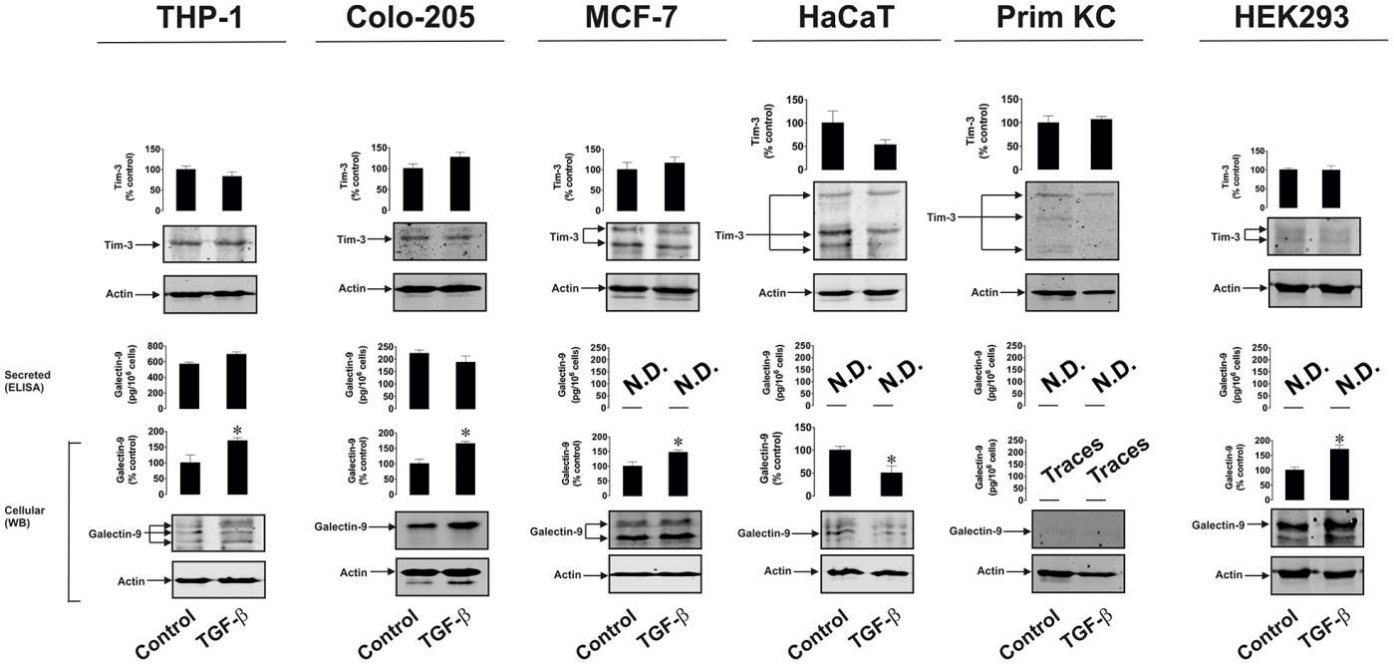
**TGF-β induces galectin-9 expression in human cancer and embryonic but not healthy cells.** THP-1 (AML), Colo-205 (colorectal cancer), MCF-7 (breast cancer) HaCaT (keratinocytes), primary human keratinocytes (Prim KC) as well as HEK293 (human embryonic kidney cells) were exposed for 24 to 2 ng/ml human recombinant TGF-β. Levels of cell-associated Tim-3 and galectin-9 as well as secreted galectin-9 were measured. Images are from one experiment representative of four which gave similar results. Data are shown as mean values ± SEM of four independent experiments.* - p < 0.05 *vs* control.

To confirm and study the differential effects of TGF-β on galectin-9 expression we treated THP-1 human AML cells, Colo205 human colorectal cancer cells, MCF-7 human breast cancer cells, HaCaT human keratinocytes (non-malignant cells), primary healthy human keratinocytes as well as HEK293 human embryonic kidney cells, with 2 ng/ml human recombinant TGF-β (specifically TGF-β type 1 was used) for 24 h. Cellular and secreted levels of galectin-9 and Tim-3 were then determined. We found that in all types of human cancer cells and in HEK293, TGF-β upregulated the amounts of expressed galectin-9 but not Tim-3. However, in non-malignant cells (both types of keratinocytes), no upregulation of either galectin-9 or Tim-3 expression was observed (Figure 8). Both types of keratinocytes expressed barely detectable levels of galectin-9 and this was not inducible by TGF-β. To find out whether such a phenomenon (the absence of induction of galectin-9 expression by TGF-β) applies also to cancer cells we used K562 chronic myeloid leukaemia cells which express only traces of galectin-9 protein [1] compared to for example THP-1 or other AML cells. Exposure of these cells to increasing concentrations of TGF-β for 24 h led to a clear induction of galectin-9 expression (Supplementary Figure 2), suggesting differential responses of cancer/embryonic and non-malignant mature human cells. Importantly, levels of phospho-S423/S425 Smad-3 were undetectable in resting K562 cells and were clearly detectable in TGF-β-treated cells (Supplementary Figure 2). Regardless the treatment, K562 cells did not release detectable amounts of galectin-9 (Supplementary Figure 2).

**Figure 9:**
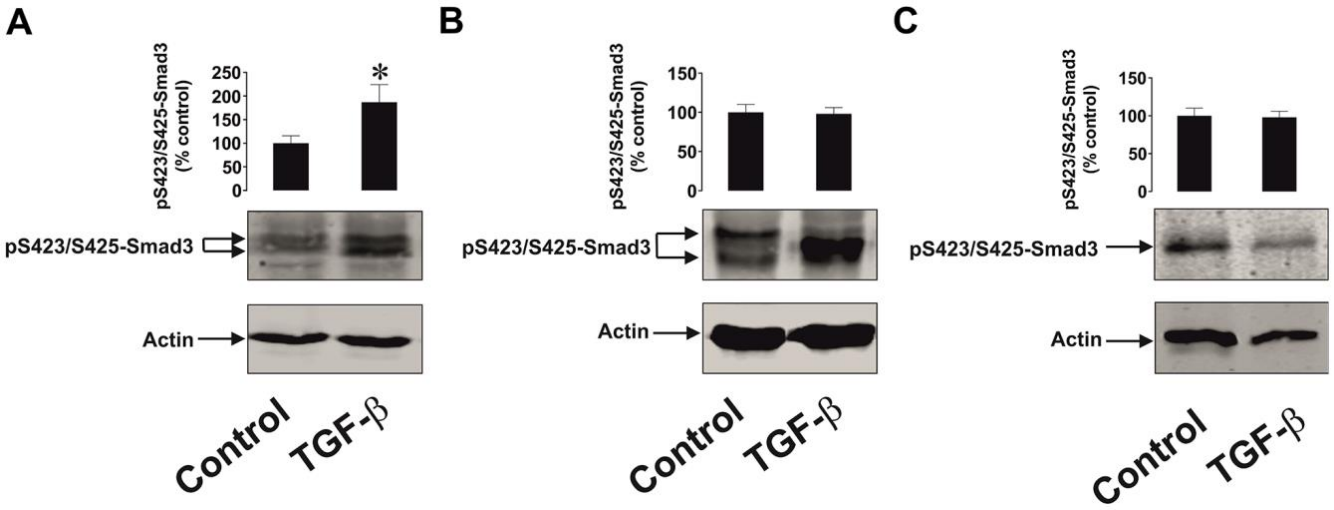
**The effects of TGF-β on Smad3 phosphorylation in human cancer and non-malignant cells.** (**A**) MCF-7 (breast cancer), (**B**) HaCaT (keratinocytes) and (**C**) primary human keratinocytes were exposed for 24 h to 2 ng/ml TGF-β followed by Western blot analysis of phospho-S423/S425-Smad3 levels. Images are from one experiment representative of four which gave similar results. Data are shown as mean values ± SEM of four independent experiments. * - p < 0.05 vs control.

Since Smad3 is the transcription factor activated by TGF-β, which then induces galectin-9 expression, we compared TGF-β-induced S423/S425 Smad3 phosphorylation in malignant and non-malignant human cells. MCF-7 breast cancer cells as well as non-malignant HaCaT cells and primary keratinocytes were exposed for 24 h to 2 ng/ml TGF-β followed by measurement of phospho-S423/S425 Smad3. We detected significant upregulation of phospho-S423/S425-Smad3 levels only in MCF-7 cells but not in non-malignant keratinocytes. In addition, the profile of the phospho-S423/S425-Smad3 band was different in malignant and non-malignant cells (Figure 9). Taken together these results suggest that TGF-β-induced Smad3-mediated galectin-9 expression takes place mainly in human cancerous and embryonic cells. The responses associated with TGF-β-induced S423/S425 Smad3 phosphorylation are clearly different in cancer/embryonic and mature non-malignant human cells.

## DISCUSSION

Galectin-9 plays a crucial role in determining the ability of cancer cells to escape host immune surveillance [1, 2]. As with all galectins, galectin-9 lacks a secretion signal sequence and thus requires trafficking in order to be externalised onto the cell surface or secreted [1, 7]. Cell surface-based or secreted galectin-9 can impair anti-cancer activities of NK and cytotoxic T cells [1, 2, 6, 7]. Tim-3 acts as a receptor and possible trafficker for galectin-9 and also participates in the transduction of moderate growth signals from galectin-9 into cancer cells (for example AML cells) as well as pro-apoptotic signals into cytotoxic T cells [3].

Many types of cancer cells express significantly higher amounts of galectin-9 compared to non-malignant cells of similar origin [1]. However, the biochemical mechanisms underlying this phenomenon remain unclear and thus investigation of galecin-9 expression control pathways was the main goal of this study.

We hypothesised that TGF-β, a growth factor with autocrine activity, is responsible for the upregulation of galectin-9 expression in cancer cells. We found that human breast tumour cells and AML cells produced significantly higher levels of TGF-β compared to non-transformed cells of similar origin. Interestingly, the levels of oxidative stress and activities of ROS producing enzymes (xanthine oxidase and NADPH oxidase) were significantly higher in cancer cells/tissues. Oxidative stress normally leads to activation of the AP-1 transcription complex [22], which contributes to TGF-β expression [25]. In addition, the levels of HIF-1α accumulation were much higher in cancer samples. HIF-1α determines transcriptional activity of the HIF-1 complex, which directly activates the expression of TGF-β. As a result of increased TGF-β activity, the levels of phosphorylated (active, when phosphorylated at S423/S425) Smad3, which is a TGF-β transcription factor, were significantly upregulated in the studied cancer cells/tissues. The levels of galectin-9 and its receptor Tim-3 were upregulated in all the studied cancer cell types (these results are shown in Figures 1 and 2). Interestingly, AML but not breast cancer patients showed significantly increased blood plasma levels of TGF-β (Figure 3), which suggests that in breast (solid) tumours TGF-β remains within tumour microenvironment, while in AML it is secreted into the blood thus having the opportunity to systemically act on AML cells in circulation.

Importantly, primary human embryonic cells showed the same pattern as breast cancer and AML cells (Figure 4). The earlier the stage the pregnancy was, the higher were the levels of galectin-9 and Tim-3 and components of the possible upstream pathway outlined above. Embryonic cells were similar to breast and other solid tumour cells and not like AML cells in terms of galectin-9 and Tim-3 secretion and where unable to secrete detectable amounts of these proteins (Figure 4).

We have confirmed that upregulation of both NADPH oxidase and xanthine oxidase are capable of increasing TGF-β production. HMGB1-induced NADPH oxidase activation led to upregulated TGF-β and galectin-9 production by THP-1 human AML cells. Blockade of NADPH oxidase activity, ASK1 kinase activity or AP-1 transcriptional activity decreased HMGB1-induced effects (Figure 5). Importantly, from our previous work we know that HMGB1 acts through Toll-like receptors (TLRs) 2 and 4 causing oxidative stress and also inducing HIF-1 activation [26]. AP-1 is known to be required for TGF-β expression although it might not directly act on the TGF-β gene [25], However, blocking AP-1 attenuates any HMGB1-induced increase in TGF-β and subsequent galectin-9 production. Specific activation of xanthine oxidase in MCF-7 human breast cancer cells also upregulated the level of oxidative burst, however it was not sufficient to induce HIF-1α accumulation (Figure 6A). Despite this, TGF-β/phospho-S423/425-Smad3 and galectin-9 levels were significantly upregulated suggesting contribution of the AP-1 pathway (Figure 6B).

We also confirmed the role of HIF-1 in TGF-β expression by exposing MCF-7 breast cancer cells to CoCl_2_, which inhibits degradative hydroxylation of HIF-1α thus causing its stabilisation, leading to HIF-1 activation. Importantly, CoCl_2_ is known to induce oxidative stress by increasing ROS generation which is achieved through acting on the mitochondrial transition pore [27, 28]. As a result, it leads to formation of free oxygen containing radicals which trigger ASK1-mediated AP-1 activation [22]. These experiments demonstrated the importance of HIF-1 in regulating TGF-β expression. While AP-1 is required but does not seem to control TGF-β gene expression directly, HIF-1 acts as a direct regulator. We found that CoCl_2_ induced TGF-β and galectin-9 expression in wild type but not in HIF-1α knockdown MCF-7 cells. This confirms the involvement of HIF-1 in CoCl_2_-induced TGF-β expression. Interestingly, in MCF-7 cells transfected with random siRNA, TGF-β expression was upregulated, although DOTAP transfection and the presence of CoCl_2_, but not TGF-β, slowed down the secretion process and galectin-9 expression was not increased, suggesting that it might depend on the autocrine activity of secreted TGF-β (Figure 7A). When studying the process in dynamics we found that CoCl_2_ rapidly induces HIF-1 activation in MCF-7 cells (after 1 h of exposure, a significant increase in HIF-1 DNA-binding activity was clearly detectable, Figure 7B). Secreted TGF-β levels were significantly increased after 3-4 h of cell exposure to CoCl_2_ whereas galectin-9 levels were only significantly upregulated after 6 h. This supports the notion that CoCl_2_-induced galectin-9 expression depends on the autocrine activity of TGF-β, the expression of which is induced by the HIF-1 transcription complex. We specifically confirmed the role of HIF-1-induced TGF-β production in upregulating the expression of galectin-9 in MCF-7 cells. Wild type MCF-7 cells were exposed to 50 μM CoCl_2_ in the absence or presence of TGF-β-neutralising antibody or isotype control antibody (Figure 7C). Since TGF-β-neutralising antibody but not isotype control attenuated CoCl_2_-induced galectin-9 expression, it demonstrates that the autocrine activity of this growth factor crucially controls the expression of galectin-9. The whole pathway includes activation of HIF-1 which upregulates TGF-β expression; TGF-β is then secreted and displays autocrine activity leading to the induction of galectin-9 expression in MCF-7 breast cancer cells. The role of Smad3 in both TGF-β self-induced expression and production of galectin-9 was confirmed using Smad3 knockdown MCF-7 cells.

Our study further demonstrated that TGF-β induces galectin-9 expression in human AML, breast and colorectal cancer as well as embryonic cells but not in the studied healthy (non-malignant) human cells. Importantly, in healthy human cells (keratinocytes) expressing barely detectable amounts of galectin-9, TGF-β cannot induce galectin-9 expression, while if cancer cells (for example K562 chronic myeloid leukaemia cells) express only traces of galectin-9, TGF-β can induce expression of this protein (Figure 8 and Supplementary Figure 2). This is in line with previous observations suggesting differential Smad3-dependent TGF-β signalling effects in malignant and non-malignant cells [17, 29]. Our investigations further confirmed that TGF-β induces S423/S425-phosphorylation of Smad3 in the studied cancer cells but not in healthy human cells. In addition, phospho-S423/S425-Smad3 Western blot band profiles vary between malignant and non-malignant human cells (Figure 9). This suggests differential responses of the investigated malignant/embryonic and non-malignant mature human cells to TGF-β in terms of their ability to react by significantly increasing galectin-9 expression. One could hypothesise that another reason for these differences in responses could be in differential reactivity of the cells in terms of TGFBR expression or their downstream signalling. Cancer/embryonic cells may retain high levels of TGFBRs on their surface while non-transformed cells may decrease these levels in response to the presence of high levels of TGF-β in the microenvironment. Another possibility is the involvement of differential co-activator proteins recruited by Smad3 in different cell types [30]. There are two main co-activators of Smad3 – transcription intermediary factor 1-gamma (TIF-1γ) also known as TRIM33 (tripartite motif-containing factor 33) and Smad4 [30]. Both co-activators and also some other binding partners (for example Smad2) are known to interact with Smad3 which influences the response Smad3 is going to trigger. In future work it will be important to understand which of co-activators/chaperons are involved in galectin-9 expression in different cell types.

Interestingly, in support of our observations, a previous clinical study has demonstrated that high expression levels of TGF-β receptors (TGFBRs) are associated with poor prognosis for AML patients and have a significant negative impact on complete remission achievement and long-term survival of these patients [31].

**Figure 10:**
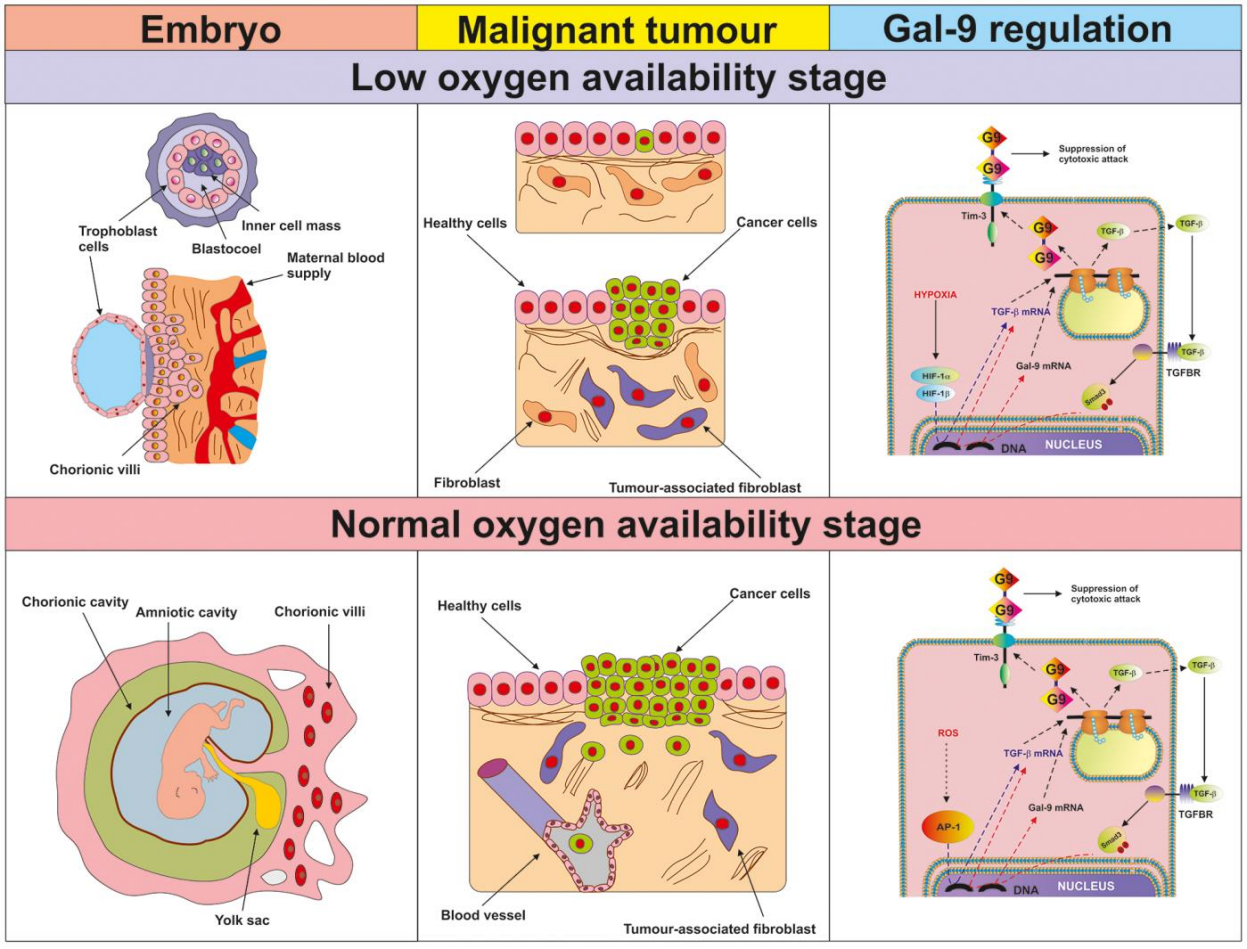
**Proposed mechanism of the regulation of galectin-9 expression in human cancer and embryonic stage at low and normal oxygen availability stages.** The figure depicts the key processes taking place in embryonic development and malignant tumour growth during the initial low oxygen availability (hypoxic) stage and later (normal oxygen availability) stages. The studied biochemical events are demonstrated in the right-hand panel. During the hypoxic stage, HIF-1 induces TGF-β expression, which then displays autocrine activity and triggers galectin-9 expression in a Smad3-dependent manner. During the normal oxygen availability stage, AP-1 contributes to TGF-β expression but it is also self-triggered by TGF-β. Galectin-9 upregulation is perpetually induced by the TGF-β-Smad3 pathway.

Our observations suggest, that during early stages of tumour growth or embryonic development, when the cells pass through a low oxygen availability stage, activation of HIF-1 induces TGF-β expression. TGF-β can then display autocrine activity and induce galectin-9 expression (a summary is shown in Figure 10). At later stages, when angiogenesis addresses the issue of low oxygen availability and normalises it, TGF-β can induce its own expression through the Smad3 transcription factor. At the same time, Smad3 can induce the expression of galectin-9 (see Figure 10). Therefore, cancer and embryonic cells studied here operate a self-supporting autocrine mechanism which is a two-stage process. During the early stage, initial TGF-β expression is, most likely, induced by the HIF-1 transcription complex and at later stages, TGF-β triggers self-expression. At both stages, TGF-β induces galectin-9 expression through the Smad3 pathway. Interestingly, TGF-β can display both tumour promoting and tumour suppressing biochemical activities [29]. However, tumour suppressive activities of the TGF-β are often avoided by tumours through acquiring mutations in critical signalling proteins or by just inhibiting TGF-β-induced anti-proliferative responses [29].

These finding demonstrate a self-supporting mechanism of galectin-9 expression operated by human AML, breast and colorectal cancer as well as embryonic cells. Our results suggest the possibility of using TGF-β signalling as a potential highly efficient target for cancer immunotherapy.

## MATERIALS AND METHODS

### Materials

RPMI-1640 cell culture medium, foetal bovine serum and supplements as well as basic laboratory chemicals were purchased from Sigma (Suffolk, UK). Microtitre plates for ELISA were obtained from Oxley Hughes Ltd (London, UK). Rabbit antibodies against galectin-9 and phospho-S423/S425-Smad3 as well as mouse antibody against HIF-1α were purchased from Abcam (Cambridge, UK). Antibodies against β-actin were purchased from Abcam (Cambridge, UK) and Proteintech (Manchester, UK). Goat anti-mouse and anti-rabbit fluorescently—labelled dye secondary antibodies were obtained from Li-COR (Lincoln, Nebraska USA). ELISA-based assay kits for the detection of galectin-9, Tim-3 and TGF-β as well as human recombinant TGF-β1 protein were purchased from Bio-Techne (R&D Systems, Abingdon, UK). Anti-Tim-3 mouse monoclonal antibody was described before [8]. All other chemicals purchased were of the highest grade of purity commercially available.

### Cell lines and primary human samples

Cell lines used in this work were purchased from either the European Collection of Cell Cultures (THP-1, Colo-205 and MCF-7), the American Tissue Culture Collection (ATTC, - HEK293) or CLS Cell Lines Service GmbH (HaCaT keratinocytes). Cell lines were accompanied by identification test certificates and were grown according to corresponding tissue culture collection protocols.

Blood plasma of healthy human donors was obtained, as previously described [9], from buffy coat blood (purchased from healthy donors undergoing routine blood donation) which was bought from the National Health Blood and Transfusion Service (NHSBT, UK) following ethical approval (REC reference: 16-SS-033). Mononuclear-rich leukocytes were isolated using Ficoll-density centrifugation according to the manufacturer's protocol [9]. Cell numbers were determined using a haemocytometer and then diluted with HEPES-buffered Tyrode's solution before treatment as indicated. Primary human AML plasma samples and cells (cultured as described before) [32] were obtained from the sample bank of University Medical Centre Hamburg-Eppendorf (Ethik-Kommission der Ärztekammer Hamburg, reference: PV3469).

Primary human breast tumour tissue samples, together with paired corresponding peripheral non-transformed tissues of the same patients, were collected through surgery from breast cancer patients at the Colchester General Hospital, following informed written consent obtained before surgery [1]. Tissue specimens were visually examined by an experienced pathologist and fresh tumour tissues were selected using conventional pathologic criteria and further confirmed by subsequent histopathological examination. Normal (non-transformed) peripheral tissues (also called “normal” or “healthy” tissues and abbreviated as HT in the figures) were selected at a distance from the site of the matching primary tumour; these tissues were microscopically inspected to confirm normal histology.

Blood samples were collected before breast surgery from patients with primary breast cancer (PBC) and before treatment of patients who had metastatic breast cancer (MBC). Samples were also collected from healthy donors (individuals with no diagnosed pathology). Blood separation was performed using a buoyancy density method employing Histopaque 1119-1 (Sigma, St. Louis, MO) according to the manufacturer's protocol [1]. Ethical approval for these studies was obtained from the NRES Essex Research Ethics Committee and the Research and Innovation Department of the Colchester Hospitals University, NHS Foundation Trust [MH 363 (AM03) and 09/H0301/37].

Placental tissues (CVS, *chorionic villus sampling*) and amniotic fluids were collected after obtaining informed written consent from pregnant women at the University Hospital Bern. Cells were prepared and propagated as described before [33]. CVS was washed with PBS, treated with 270 U/ml of collagenase type 2 (Sigma, Buchs, Switzerland) for 50 min at 37° C, washed twice with PBS and cells were then re-suspended and cultured in CHANG medium (Irvine Scientific, Irvine, USA) according to the manufacturer’s instructions. Amniotic fluid samples were centrifuged and cell pellets were then re-suspended in CHANG medium. The first medium change was performed after 5 days of incubation at 37° C. The medium was then changed every second day until the number of cells was sufficient.

Primary keratinocytes from cleft lip palate patients were cultured in keratinocyte medium as described previously [34]. Briefly, fresh tissue samples were placed into sterile tubes containing Dulbecco’s modified Eagles medium (DMEM, Gibco/Life Technologies; Thermo Fischer Scientific, Lucerne, Switzerland) supplemented with 10% FCS. The tissue was chopped into small pieces (< 1 mm^3^) and placed into 6-well plates in 800 μl DMEM, 10% FCS, 1xAmphotericin B. In mixed cell-type outgrowths, fibroblasts were separated from keratinocytes by differential trypsinization. Keratinocytes were then cultured in keratinocyte basal serum-free medium (KSFM, Gibco), supplemented with 25 mg/ml bovine pituitary extract, 0.2 ng/ml epidermal growth factor, and CaCl_2_ to a final concentration of 0.4 mM, as previously described [35]. Primary cells were tested for their purity by qPCR and immunofluorescent staining [34]. Isolation of human cells was approved by the Kantonale Ethikkommission of Bern, Switzerland, protocol number 2017-01394). Written informed consent was obtained from the parents of the children involved.

### Plasmids

Plasmid encoding hemagglutinin (HA)-tagged human ASK1 with kinase-dead domain (dominant-negative form), ΔN-ASK1, was a kind gift of Professor Ichijo (University of Tokyo, Tokyo, Japan). Plasmid was amplified using E. Coli XL10 Gold® (Stratagene Europe, Amsterdam, The Netherlands) and isolated/purified using the GenElute™ plasmid purification kit according to the manufacturer's protocol. Purified plasmids were transfected into THP-1 cells using DOTAP transfection reagent according to the manufacturer's protocol [24].

### Transfection of HIF-1α siRNA into MCF-7 cells

siRNA specific to HIF-1α was selected as described previously and purchased from Sigma (Suffolk, UK) together with a mutated form of siRNA (called random siRNA, which was used as negative control) [24]. We employed a HIF-1α-specific siRNA target sequence (ugu gag uuc gca ucu uga u dtdt) localised at position 146 bases downstream of the HIF-1α start codon. Smad3 siRNA was a commercially available reagent purchased from Ambion (ID 107876) through Thermo Fisher Scientific. Random (mutated) siRNA used as a negative control in all the knockdown experiments had the following sequence: uac acc guu agc aga cac c dtdt. siRNAs were transfected into THP-1 cells using DOTAP transfection reagent according to the manufacturer's protocol. Successful HIF-1α knockdown was verified by assessing HIF-1 DNA-binding activity.

### Western blot analysis

Galectin-9, Tim-3, HIF-1α and phospho-S423/S425 Smad-3 were measured by Western blot and compared to the amounts of β-actin (protein loading control), as previously described [1]. Cells were lysed in 50 mM Tris–HCl, 5 mM EDTA, 150 mM NaCl, 0.5% Nonidet-40, 1 mM PMSF, pH 8.0. Tissue lysates for Western blot analysis were prepared as described previously. Briefly, 100 mg of frozen tissues were grounded into a powder in dry ice, followed by the addition of 100 μl of tissue lysis buffer (20 mM Tris/HEPES pH 8.0, 2 mM EDTA, 0.5 M NaCl, 0.5% sodium deoxycholate, 0.5% Triton X-100, 0.25 M sucrose, supplemented with 50 mM 2-mercaptoethanol, 50 μM PMSF, and 1 μM pepstatin which was supplied just before use). Tissues were homogenised using a Polytron homogenizer (Capitol Scientific, USA) and a syringe was applied in order to acquire a homogenous tissue suspension. These tissue suspensions were then filtered through medical gauzes and centrifuged at +4° C at 10,000 g for 15 min. Proteins present in supernatants were precipitated by incubation of the samples on ice for 30 min with equal volumes of ice-cold acetone. Protein pellets were obtained by centrifugation at +4° C, 10,000 g for 15 min followed by air drying at room temperature and then mixed with the SDS-lysis buffer described above. When measuring transcription factors, cell lysis buffer described above was also applied.

Li-Cor goat secondary antibodies conjugated with infrared fluorescent dyes, were used as described in the manufacturer's protocol in order to visualise specific proteins (Li-Cor Odyssey imaging system was employed). Western blot data were quantitatively analysed using Odyssey software and values were subsequently normalised against those of β-actin or total protein loaded.

### Detection of HIF-1 DNA-binding activity

HIF-1 DNA-binding activity was measured using the method similar to the one we recently described, with some modifications [36]. A 96-well Maxisorp™ microtitre plate was coated with streptavidin and blocked with BSA as described before. A volume of 2 pmol/well biotinylated 2HRE-containing oligonucleotide was immobilised by 1 h incubation at room temperature. The plate was then washed five times with TBST buffer (10 mM Tris-HCl, pH 8.0, 150 mM NaCl, 0.05% Tween-20), followed by 1 h incubation with 20 μl/well of cell lysate at room temperature. The plate was again washed with TBST buffer and mouse anti-HIF-1α antibody (1:1 000 in TBS with 2% BSA) was added. After 1 h of incubation at room temperature the plate was washed with TBST buffer and then incubated with 1:1 000 Li-Cor goat anti-mouse secondary antibody labelled with infrared fluorescent dye. After extensive washing with TBST, the bound secondary antibody was detected using Li-Cor fluoroimager.

### Enzyme-linked immunosorbent assays (ELISAs)

Secreted TGF-β, galectin-9 and Tim-3 were measured, either in cell culture medium or in blood plasma, by ELISA using R&D Systems kits according to manufacturer’s protocols.

### Detection of xanthine oxidase and NADPH oxidase activities as well as quantitation of thiobarbiturate reactive species (TBRS)

Xanthine oxidase activity was measured using a spectrophotometric assay described previously [21]. NADPH oxidase activity was measured based on the ability of this enzyme to produce superoxide anion radical [36]. TBRS were quantified using a previously described colorimetric assay [37].

### Statistical analysis

Each experiment was performed at least three times and statistical analysis, when comparing two normally distributed events at a time, was conducted using a two-tailed Student's *t*-test. In cases when multiple comparisons (more than two groups) were performed, we used an ANOVA test. Post-hoc Bonferroni correction was used where applicable. Statistical probabilities (p) were expressed as * when p<0.05; **, p<0.01 and *** when p<0.001.

## Supplementary Material

Supplementary Figures
