# Supplementary material for: Transforming growth factor beta type 1 (TGF-β) and hypoxia-inducible factor 1 (HIF-1) transcription complex as master regulators of the immunosuppressive protein galectin-9 expression in human cancer and embryonic cells
Source: Aging (Albany NY). 2020 Dec 8;12(23):23478–96. doi: 10.18632/aging.202343 (PMC7762483; doi:10.18632/aging.202343)
Supplement: Supplementary Figures [file aging-12-202343-s001.pdf]

SUPPLEMENTARY FIGURES

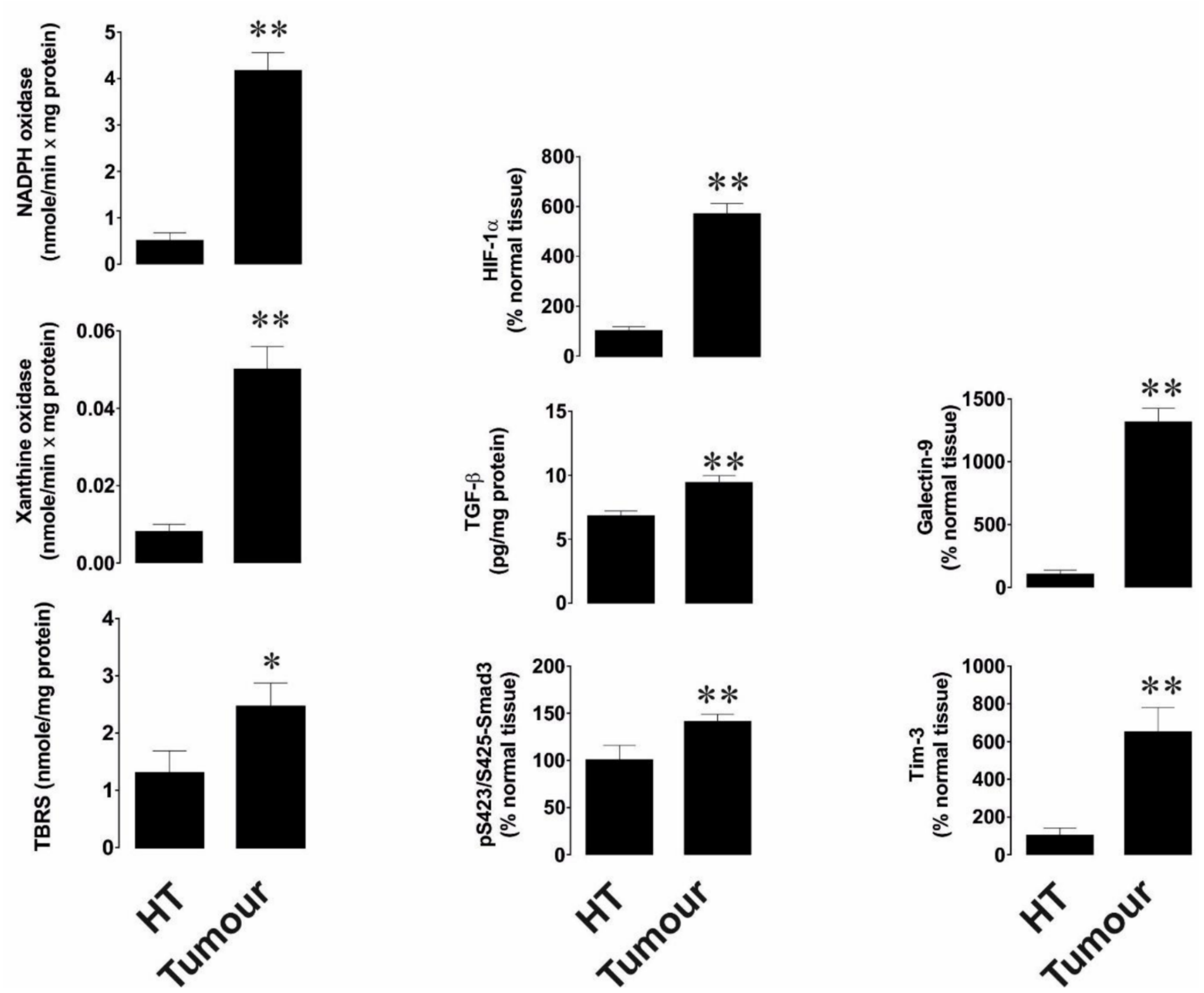

Supplementary Figure 1. Values presented in Figure 1 normalised against total protein loaded for Western blot data and per 1 mg of total tissue protein for enzyme activity and TBRS assays. Data are shown as mean values ± SEM of five independent experiments. * - $p < 0.05$ and ** - $p < 0.01$ *vs* non-transformed peripheral tissue abbreviated as HT (healthy tissue).

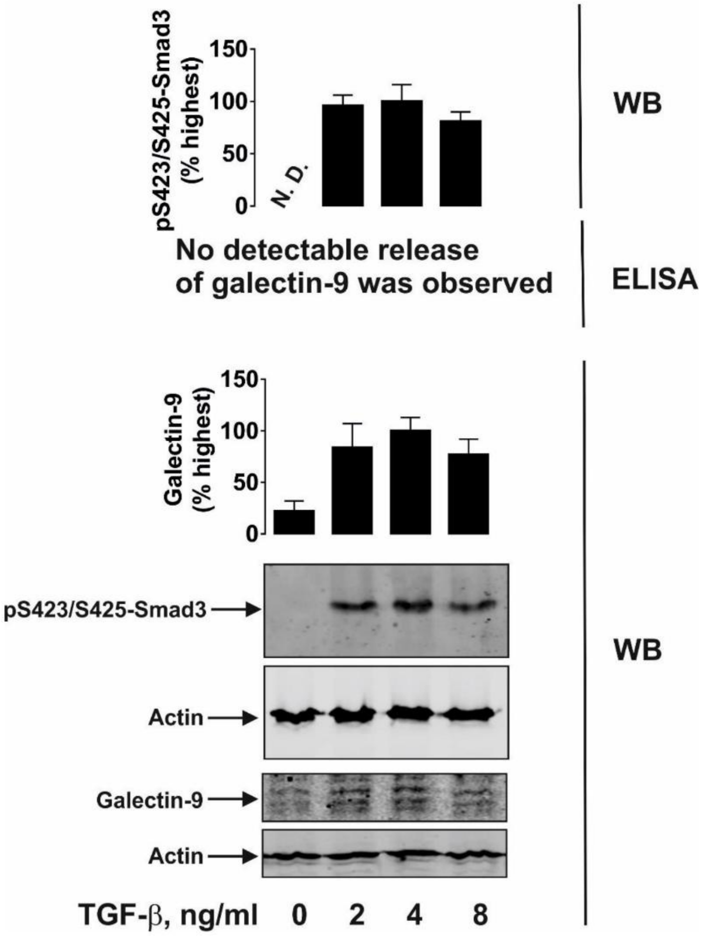

Supplementary Figure 2. TGF-β induces galectin-9 expression in K562 human cancer cells. K562 human chronic myeloid leukaemia cells were exposed for 24 h to 2, 4 or 8 ng/ml TGF-β followed by detection of phospho-S423/S425-Smad3 and galectin-9 expression by Western blot. Galectin-9 release was analysed by ELISA. Images are from one experiment representative of three which gave similar results. Data are shown as mean values ± SEM of three independent experiments.
